# ﻿A new species of *Argyreia* (Convolvulaceae) from Yunnan, China

**DOI:** 10.3897/phytokeys.225.100646

**Published:** 2023-05-02

**Authors:** Mao-Lin Zhang, Yi He, Quan-Ru Liu

**Affiliations:** 1 Key Laboratory of Biodiversity Science and Ecological Engineering, Ministry of Education, College of Life Sciences, Beijing Normal University, Beijing 100875, China Beijing Normal University Beijing China

**Keywords:** *Argyreiasubrotunda*, flora of Yunnan, morphology, new taxon, taxonomy

## Abstract

*Argyreiasubrotunda*, a new species from Yunnan Province, China, is described and illustrated. The new species resembles *A.fulvocymosa* and *A.wallichii*, but differs from these in the flowers with an entire or shallowly lobed corolla, as well as smaller elliptic bracts, lax flat-topped cymes and shorter corolla tubes. An updated key to the species of *Argyreia* from Yunnan province is also provided.

## ﻿Introduction

*Argyreia* Lour., a genus comprising scandent shrubs or lianas, is mainly distributed throughout tropical Asia (Staples and Traiperm 2017). *Argyreia* species mainly inhabit open and sunny places such as roadsides, thickets, and edges of mingled forest (Fang and Huang 1979; Fang and Staples 1995). The number of *Argyreia* species has been increasing and is now up to 143 species following the discovery of new species (Traiperm et al. 2019; Traiperm and Suddee 2020) and the establishment of new combinations (Shalini et al. 2017; Staples and Traiperm 2017; Rattanakrajang et al. 2022). There are about 25 species in China (14 of which are endemic) and 92% of the species found in China in Yunnan Province (23 recorded species). The province of Yunnan is, therefore, the main center of diversity in China (Fang and Huang 1979; Fang and Staples 1995; Yang et al. 2015).

Loureiro (1790) published *Argyreia* as a genus within Convolvulaceae. The genus is mainly characterized by indehiscent fleshy or mealy berries (Staples and Traiperm 2017). The various types of indumentum, inflorescence architecture, depth of corolla lobes, and number of seeds in individual berries are the main taxonomically informative characters for the delimitation of species in *Argyreia*.

*Argyreia* seems to be non-monophyletic in recent works, because it includes at least one of the moth-pollinated species of *Rivea* Choisy (Manos et al. 2001; Stefanovic et al. 2003). Furthermore, there is evidence that *Blinkworthia* Choisy should be subsumed under *Argyreia* (Rattanakrajang et al. 2022). We support this conclusion and although the *Argyreia* alliance clade as recovered is paraphyletic, only one *Rivea* species was used and the inclusion of other species might lead to different conclusions in the future (Rattanakrajang et al. 2022). Therefore, we think that *Rivea* and *Argyreia* are two independent genera and both supposedly monophyletic, but their limits should be revised under a phylogenetic perspective with a comprehensive sampling.

Although recent studies have shown that *Argyreia* should be treated as part of *Ipomoea* L. (Muñoz-Rodríguez et al. 2019) and *Argyreia* is merged into *Ipomoea* by Wood et al. (2020), we chose not to follow the proposed classification in the present work, as further study of Old World taxa is still required (Traiperm and Suddee 2020). As it concerns *Ipomoea*, the possibility to keep the several established smaller genera has the potential to maintain nomenclatural stability (Eserman et al. 2020). So we do not subsume *Argyreia* into *Ipomoea* at this time and accordingly maintain the well-established generic concepts (Rattanakrajang et al. 2022).

During recent field surveys in Yunnan Province, an interesting population of *Argyreia* with an entire or shallowly lobed corolla was found. After reviewing literature and comparing specimens, especially native species in Yunnan Province and adjacent countries (Vietnam, Laos, Myanmar, Cambodia and Thailand), we found that the taxon was not completely similar to any species known worldwide. Therefore, a new *Argyreia* species from China is described and illustrated here.

## ﻿Material and method

Plant material was collected during field surveys in Yunnan Province from 2020 to 2021. The type specimens have been stored in the herbarium of Beijing Normal University (**BNU**). Morphological measurements were made from dried specimens of herbarium by Nikon digital camera, Stereoscope (ZEISS V8) and software ImageJ (Abràmoff et al. 2004). Materials for observation of pollen morphology were obtained from herbarium, picking mature pollen from the dried specimens, sticking it on the sample stages with conductive adhesive, spraying gold and photographing by Scanning Electron Microscope. The collected specimens were compared with the type specimens of morphologically similar species at main herbariums in China (BNU, HITBC, IBSC, KUN, PE, WUK, YUKU), as well as digital images available online provided by JSTOR and herbaria abroad that are relevant for the group (A, BM, E, G, K, P). Fresh plant materials of the similar species (*A.wallichii*) were also collected for further careful comparison. All type specimens (or photos of type specimens) of accepted names and their synonyms in *Argyreia* known around the world were examined, which refer to the voucher information provided by Staples and Traiperm (2017).

## ﻿Taxonomic treatment

### ﻿ Argyreia subrotunda

Taxon classificationPlantaeSolanalesConvolvulaceae

Q.R.Liu & M.L.Zhang sp. nov.

4B08EF65-8DC3-503A-AEC0-BAB7CC79B2B4

urn:lsid:ipni.org:names:77318528-1

Figs 1 , 2

#### Type.

China. Yunnan Province: Malipo County, Xinzhai Village, 22°57'48.01"N, 104°46'31.11"E, along roadside, 1300 m elev., 27 Aug 2021, fl. *M. L. Zhang BNU2021YN074* (holotype: BNU0053319!; isotypes: BNU!).

**Figure 1.**
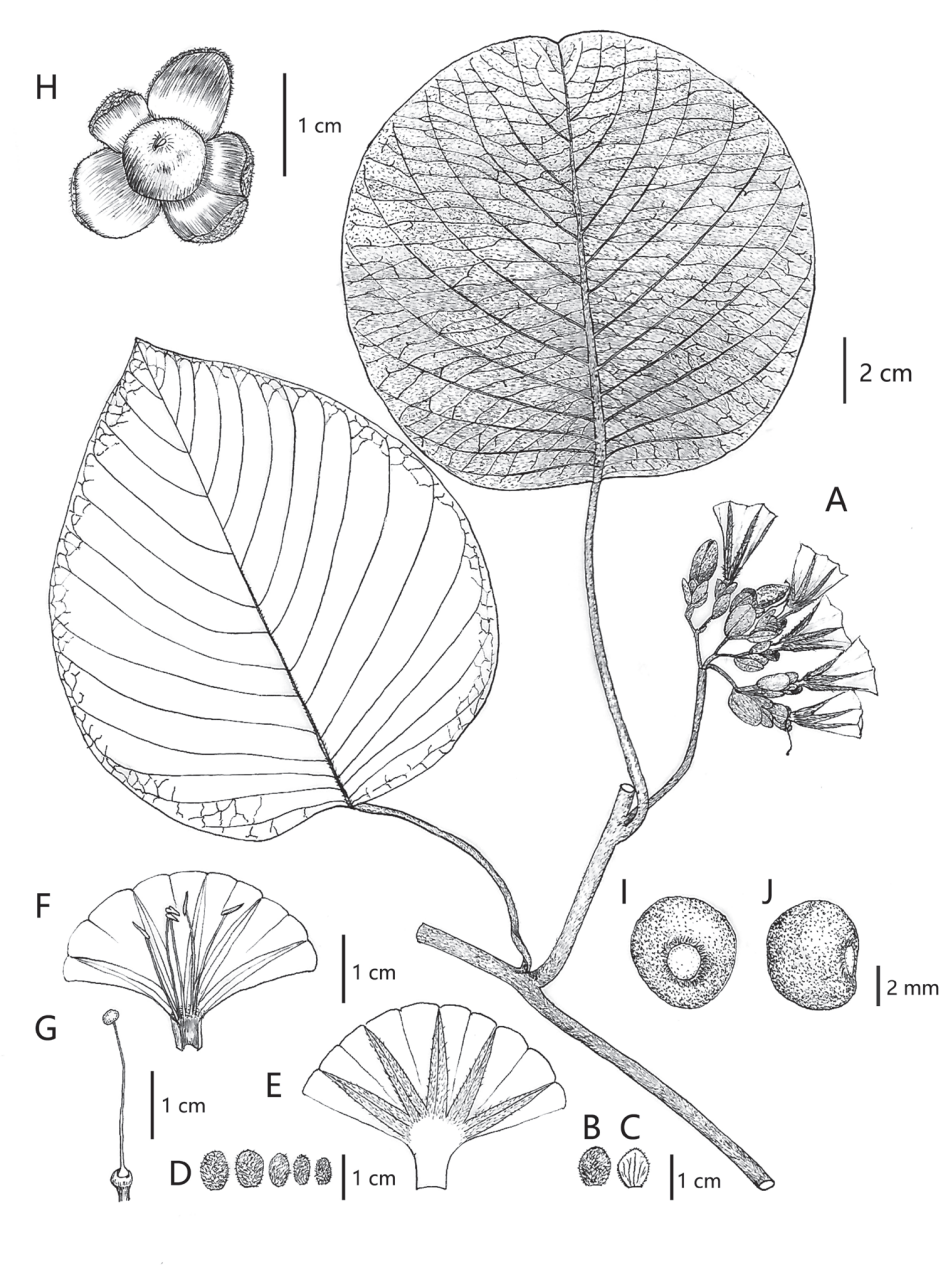
*Argyreiasubrotunda* Q.R.Liu & M.L.Zhang, sp. nov. **A** stem with leaves and inflorescences **B** bract (outside) **C** bract (inside) **D** sepals from outer (left) to innermost (right) **E** opened corolla showing mid-petaline bands **F** opened corolla with stamens **G** pistil **H** fruit with persistent sepals **I** seed (adaxial surface) **J** seed (lateral surface). All drawn by Quan-Ru Liu from voucher specimens *M. L. Zhang BNU2021YN074* (BNU!) (**A–G**), *X. B. Guo BNU2021YN081* (BNU!) (**H–J**).

#### Diagnosis.

*A.subrotunda* is unique, a small-flowered type with an entire or shallowly lobed corolla as well as exserted stamens and pistils (included in dry specimens), smaller elliptic bracts, and outer sepals ovate-circular. It is similar to *A.wallichii* in indumentum features (whitish tomentose) and fruit types (red globose berry), but differs by its smaller elliptic bracts (vs. ovate-oblong), lax flat-topped cymes (vs. compact capitate) and shorter corolla tubes (2–2.5 cm vs. 4–5 cm). Additionally, *A.subrotunda* is similar to *A.fulvocymosa* in leaf shape (broadly ovate-circular to nearly circular) and inflorescence (flat-topped cymes), but the latter is covered with densely yellowish villus and has a distinctly 5-lobed corolla, which is very easy to distinguish (Table 1).

**Table 1.** Comparisons of *A.fulvocymosa*, *A.subrotunda* and *A.wallichii*.

Character		*A.fulvocymosa*	*A.subrotunda*	*A.wallichii*
Inflorescence		flat-topped cymes	**flat-topped cymes**	**capitate cymes**
Bract	Shape	unknown	**elliptic**	**ovate-oblong**
	Size	unknown	**0.8–1** cm × **0.4–0.8** cm	**2.5–3.5** cm × **1.5–2.5** cm
Outer sepal	Shape	broadly ovate-circular	ovate-circular	elliptic-oblong
Corolla	Length	ca. 2 cm	**2–2.5** cm	**4–5** cm
	Mid-petaline bands indumentum	yellowish hirsute	whitish villous	whitish villous
	Limb	**distinctly 5-lobed**	**entire or shallowly lobed**	entire or shallowly lobed
Stamen and pistil		exserted	exserted	included

#### Description.

Climbing lianas; stem woody at base, herbaceous above, the former puberulent, the latter covered with whitish trichomes. Leaves simple, alternate; petiole 6–10 cm long, tomentose; leaf blades broadly ovate to rounded, 13–16 × 12–15 cm; base truncate or slightly cordate, occasionally oblique, margins entire, apex acute or obtuse, sometimes slightly emarginate; adaxially green, sparsely whitish velutinous only along leaf veins, abaxially paler, densely shining tomentose; secondary veins 13–15 on either side, curved to edge, veins slightly raised adaxially, more prominently raised abaxially. Inflorescences flat-topped cymes, axillary or terminal; peduncle 2–5 cm long, tomentose, angulate, secondary and tertiary peduncle 6–12 mm long; bracts small, elliptic, 8–10 × 4–8 mm, obtuse, hairy outside, veined; pedicels 5–7 mm long, up to 10 mm in fruit. Flowers diurnal; sepals unequal, 2 outer ovate-circular, 8–9 × 6–7mm, 3 inner elliptic, 6–7 × 3–5 mm, apex obtuse, abaxially whitish tomentose, adaxially glabrous, veined, enlarged in fruit, rose-red, shiny. Corolla tubular-funnelform, 2–2.5 cm long, pink, densely whitish villous outside on mid-petaline bands, otherwise glabrous, limb entire or shallowly lobed. Stamens exserted; filaments filiform, 14–15 mm long, attaching to the site of ca. 5 mm from stamens base, expanded at attachment points and densely whitish hairy there; anthers oblong, 3–4 mm long; pollen globose, pantoporate, with spines, 90–101 μm in diameter. Pistil exserted; disc ringlike, glabrous, ca. 1 mm high; ovary glabrous, ovoid, 2–3 mm high; style filiform, glabrous, 20–22 mm long; stigmas capitate, 2-lobed. Fruit enclosed in persistent, accrescent calyx, 2 outer fruiting sepals enlarging to 10–11 × 7–8 mm, 3 inner sepals 8–10 × 5–6 mm; berry subglobose, 7–10 mm in diam., purple-red, glabrous, exocarp leathery shiny, with obvious stomata under a magnifier, wrinkled when dry. Seeds 1–2, subglobose or hemispherical, 3.5–4 × 4–4.5 × 2.5–3 mm, black, glabrous, surface not smooth; hilum subcordate, brown, basal, margin with sparsely whitish hairy.

**Figure 2.**
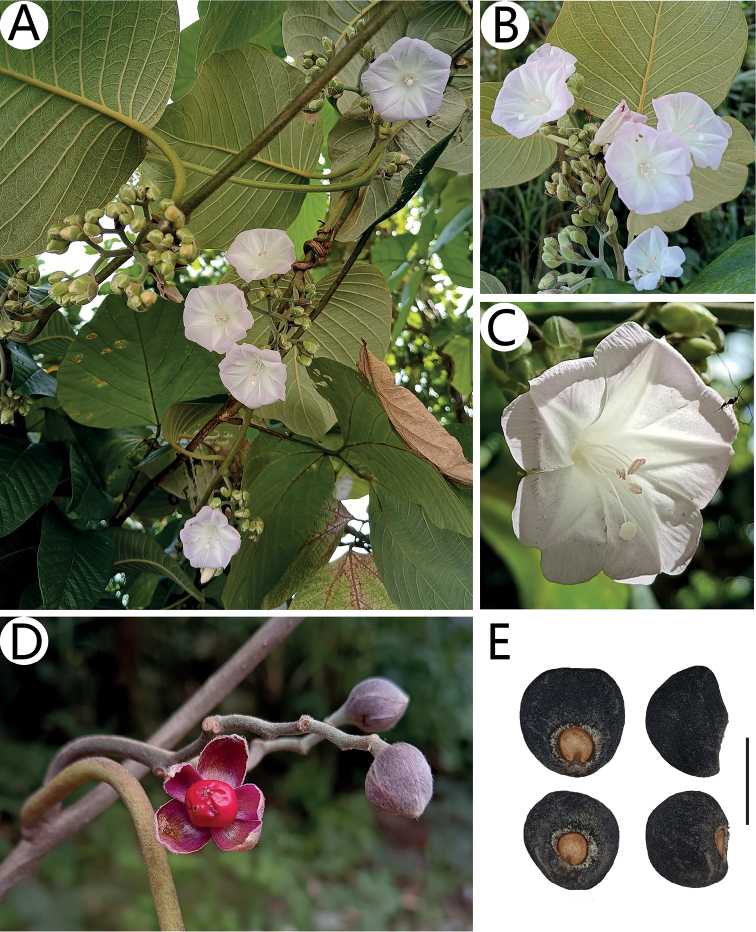
*Argyreiasubrotunda* Q.R.Liu & M.L.Zhang, sp. nov. **A** plant habit **B** inflorescence **C** flower in frontal view **D** fruit with persistent sepals **E** seeds: adaxial surface (left); lateral surface (right). Scale bar: 5 mm. Photographs **A–C, E** by Mao-Lin Zhang, **D** by Xi-Bing Guo.

#### Phenology.

Flowering from August to November; fruiting in November to February.

#### Distribution and habitat.

**Figure 3.**
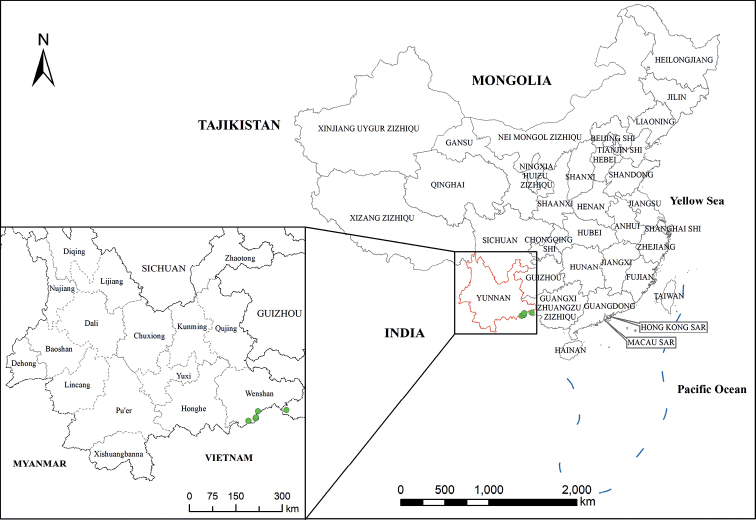
Distribution of *Argyreiasubrotunda* in China. Drawn by Yi He.

Distributed in Yunnan and Gaungxi Province (Fig. 3), occurring at elevations of ca. 650–1300 m, distributed at open and sunny places such as roadsides, thickets, edges of mingled forest.

#### Preliminary conservation status.

Least Concern (LC). At present, five populations have been collected in Malipo County, Maguan County and Napo County. Each population is large with high flowering rates, and the number of mature individuals in the population is more than 50. According to the IUCN (2019) red list categories and criteria, *A.subrotunda* should be categorized as a ‘Least Concern (LC)’ species, which needs further investigation and research to more fully assess the conservation status.

#### Etymology.

The specific epithet refers to the leaf shape, which is near-round.

#### Chinese name.

近圆叶银背藤 (Jìn Yuán Yè Yín Bèi Téng).

#### Additional specimens examined.

China, Guangxi Province: Napo County, Baisheng Township, Naen Reservoir, 26 Nov. 2013, fr. *B. Y. Huang et al. 451026131126017LY* (GXMG!); Yunnan Province: Malipo County, Bar-bu, 1000 m elev., 2 Feb. 1940, fr. *C. W. Wang et al. 86509* (PE!); Malipo County, Wen-tian Road beside National Highway G246, 650 m elev., 23 Nov. 2021, fr. *X. B. Guo BNU2021YN081* (BNU!); Malipo County, Xinzhai Village, 1300 m elev., 23 Nov. 2021, fl. *X. B. Guo BNU2021YN082* (BNU!).

##### ﻿Pollen morphology

**Figure 4.**
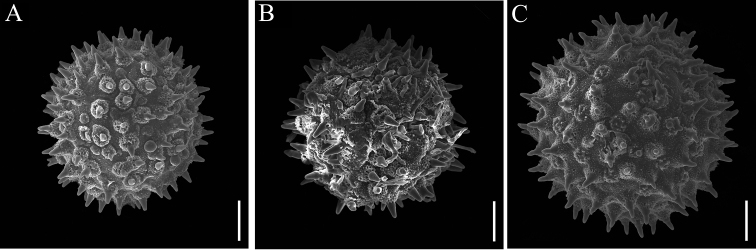
Comparison of pollen morphology **A***Argyreiasubrotunda***B***A.wallichii***C***A.marlipoensis*. Scale bars: 20 μm.

The observed pollen grains of *A.subrotunda* were monad, spheroidal to subspheroidal and radially symmetrical, with polypantoporate and echinate ornamentation (Fig. 4). It was possible to divide into two types based on the pollen morphology as follows: the diameter of the pollen grain was less than 100 μm with shorter bottle-like spines (5–7 μm), such as *A.wallichii* and the new species *A.subrotunda*; the diameter of pollen grains was over 100 μm with longer cone-shaped spines (≥ 10 μm), such as *A.marlipoensis*, which is endemic to Yunnan province, and the flower of which is first seen in this study.

## ﻿Discussion

**Figure 5.**
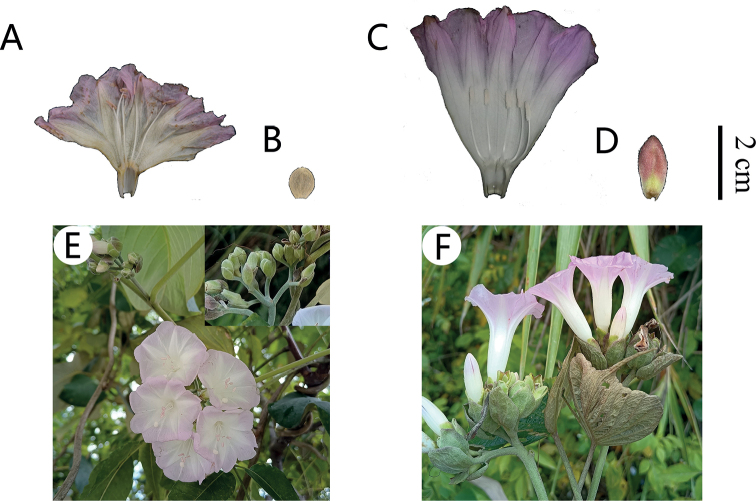
*Argyreiasubrotunda***A** opened corolla with 5 stamens **B** bract **E** inflorescence. *A.wallichii***C** opened corolla with 5 stamens **D** bract **F** inflorescence.

Morphologically, this species is most similar to *A.wallichii* and *A.fulvocymosa*, and it can be easily distinguished by the characters summarized in Table 1. The new species was similar to *A.wallichii*, both having similar indumentum features and fruit types as well as being almost sympatric. However, based on 17 specimens from two populations of *A.subrotunda*, we found the length of corolla was a very stable feature, about 2–2.5 cm, which was significantly shorter than *A.wallichii* (4–5 cm). The latter could also be easily distinguished from the new species by its compact capitate cymes and ovate-oblong bracts instead of flat-topped cymes and elliptic bracts. Additionally, *A.subrotunda* was similar to *A.fulvocymosa* in leaf shape and inflorescence, but the latter had a distinctly 5-lobed corolla instead of an entire or shallowly lobed corolla. Morphological comparisons of fresh plants between *A.subrotunda* and *A.wallichii* were provided in Figure 5. Furthermore, detailed comparisons of *A.fulvocymosa*, *A.subrotunda* and *A.wallichii* were provided in Table 1.

The discovery of the new species has important value for further understanding of the morphological patterns of *Argyreia* in China. The new species is endemic to southwest China and compared to other species with an entire or shallowly lobed corolla in China, it seems to have comparative smaller corollas, which reinforces its recognition as a new taxa.

### ﻿Key to the species of *Argyreia* from Yunnan, China

**Table:** 

1	Corolla entire or shallowly lobed	**2**
–	Corolla deeply 5-lobed	**17**
2	Climbing herb, stems slender, roots thick	**3**
–	Climbing shrubs or lianas, stem woody at base	**4**
3	Leaf blade linear; sepals ovate; corolla pale purple, 4–4.5 cm long	***A.lineariloba***
–	Leaf blade ovate to ovate-deltate; sepals linear-lanceolate; corolla red, ca. 7 cm long	***A.baoshanensis***
4	Bracts soon deciduous	**5**
–	Bracts persistent	**7**
5	Leaf blade densely yellowish sericeous-velutinous abaxially	***A.velutina***
–	Leaf blade sparsely strigose or hispid abaxially	**6**
6	Leaf blade lanceolate or ovate to ovate-elliptic	***A.henryi***
–	Leaf blade broadly ovate to nearly circular, truncate or slightly cordate	***A.strigillosa***
7	Bracts with more than 15 mm in length	**8**
–	Bracts 2–13 mm in length	**13**
8	Inflorescence paniculate-umbelliform, lax	**9**
–	Inflorescence capitate, condensed	**10**
9	Leaf blade sparsely hispid abaxially, somewhat reddish purple colored; bracts narrowly lanceolate; corolla campanulate-funnelform, 5–7 cm long	***A.marlipoensis***
–	Leaf blade densely silvery sericeous; bracts ovate-circular; corolla urceolate-funnelform, 2.5–3.5 cm long	***A.monosperma***
10	Indumentum brown or dull yellow, hirsute	***A.capitiformis***
–	Indumentum whitish or pale yellow, villous or pubescent	**11**
11	Bracts ligulate, petiolate, apex acuminate	**A.roxburghiivar.ampla**
–	Bracts ovate-circular or broadly ovate, apex acute or obtuse	**12**
12	Bracts densely curly sericeous villous abaxially, ovate-circular; sepals brown, narrow-oblong	***A.eriocephala***
–	Bracts densely pubescent abaxially, broadly ovate; sepals rose purplish, ovate-oblong	***A.wallichii***
13	Leaf blade broadly ovate-circular, base truncate or slightly cordate, densely yellowish villous or whitish tomentose abaxially	**14**
–	Leaf blade narrowly oblong or ovate-oblong, to elliptic, base rounded or broadly cuneate, densely silvery sericeous-pilose abaxially	**15**
14	Leaf blade densely dull yellowish sericeous villous abaxially; corolla broadly funnelform, ca. 4.5 cm long, purple	***A.fulvovillosa***
–	Leaf blade densely whitish tomentose abaxially; corolla tubular-funnelform, 2–2.5 cm long, pink	***A.subrotunda***
15	Peduncle 10.5–13.5 cm long	***A.splendens***
–	Peduncle less than 10 cm long	**16**
16	Sepals subequal, ovate-oblong; corolla white	***A.cheliensis***
–	Sepals unequal, elliptic or oblong; corolla purple	***A.monglaensis***
17	Inflorescence capitate; bracts persistent; peduncle short to none, 0–0.3 cm long	***A.osyrensis***
–	Inflorescence paniculate-umbelliform; bracts tiny or caducous; peduncle elongated, ca. 0.5 cm long	**18**
18	Young stems, leaves abaxially, inflorescence all densely silvery sericeous-pilose; cymes axillary or terminal, 3–10-flowered	***A.obtusifolia***
–	Young stems, leaves abaxially, inflorescence all densely yellowish tomentose; cymes axillary, 9–40-flowered	***A.fulvocymosa***

## Supplementary Material

XML Treatment for Argyreia subrotunda
